# Supplementary material for: Pterostilbene, a Dimethyl Derivative of Resveratrol, Exerts Cytotoxic Effects on Melanin-Producing Cells through Metabolic Activation by Tyrosinase
Source: Int J Mol Sci. 2024 Sep 17;25(18):9990. doi: 10.3390/ijms25189990 (PMC11432345; doi:10.3390/ijms25189990)

SUPPORTING INFORMATION

Additional Supporting Information may be found online in the supporting information tab for this article.

Table S1. Assignments of signals from ^{1}H and ^{13}C NMR spectra (in CD_3OD) of DiNAC-PTS-catechol (**5**) and TriNAC-PTS-catechol (**6**).

Figure S1. ^{1}H NMR spectrum (in CD_3OD) of TriNAC-PTS-catechol (**6**). A signal for <u>H</u>COOH appeared at 8.07 ppm.

Figure S2. ^{1}H NMR spectrum (in CD_3OD) of DiNAC-PTS-catechol (**5**). A signal for <u>H</u>COOH appeared at 8.07 ppm.

Figure S3. ^{1}H NMR spectrum (in CD_3OD) of PTS-catechol (**4**).

Figure S4. ^{13}C-NMR spectrum (in CD_3OD) of DiNAC-PTS-catechol (**5**).

Figure S5. ^{13}C-NMR spectrum (in CD_3OD) of TriNAC-PTS-catechol (**6**).

Figure S6. ^{13}C-NMR spectrum (in CD_3OD) of PTS-catechol (**4**).

Figure S7. 2D DQF-COSY of DiNAC-PTS-catechol (**5**).

Figure S8. 2D DQF- COSY of TriNAC-PTS-catechol (**6**).

Figure S9. 2D DQF-COSY of PTS-catechol (**4**).

Figure S10. 2D HMBC of DiNAC-PTS-catechol (**5**).

Figure S11. 2D HMBC of TriNAC-PTS-catechol (**6**).

Figure S12. 2D HMBC of PTS-catechol (**4**).

Figure S13. 2D HMQC of DiNAC-PTS-catechol (**5**).

Figure S14. 2D HMQC of TriNAC-PTS-catechol (**6**).

Figure S15. 2D HMQC of PTS-catechol (**4**).

Figure S16. Tyrosinase-dependent cytotoxicity against **(a)** PTS (**2**), **(b)** RES (**1**), and **(c)** 4SCAP in human tyrosinase-expressing 293T cells (hTYR-293T cells). Viability of TYR- or mock-transfected cells treated with the indicated concentrations of compounds for 2 h were assessed. Data represent means ± SD (n = 3 wells). Similar results were obtained in two or three experiments.

Table S1. Assignments of signals from ^{1}H and ^{13}C NMR spectra (in CD_3OD) of DiNAC-PTS- catechol (**5**) and TriNAC-PTS-catechol (**6**). Chemical shifts were referenced to the solvent signals (3.30 ppm for ^{1}H or 49.0 ppm for ^{13}C).

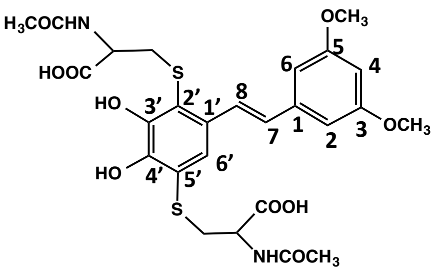

DiNAC-PTS-catechol (**5**)

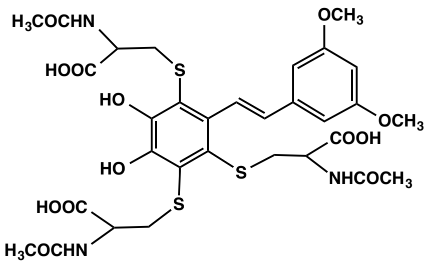

TriNAC-PTS-catechol (**6**)

Position	DiNAC-PTS-catechol (**5**)			TriNAC-PTS-catechol (**6**)		
	δ_H	Mult., *J* (Hz)	δ_C	δ_H	Mult., *J* (Hz)	δ_C
1			141			141
2	6.73	d, J = 2.0	106	6.72	d, J = 2.0	106
3			162			163
4	6.40	dd, J = 2.2, 2.4	101	6.41	dd, J = 2.2, 2.2	101
5			162			163
6	6.73	d, J = 2.0	106	6.72	d, J = 2.0	106
7	6.97	d, J = 16.4	130	6.55	d, J = 16.0	129
8	7.71	d, J = 16.0	128	7.34	d, J = 16.4	127
1′			134			137
2′			123			121
3′			148			142
4′			146			142
5′			123			121
6′	7.35	s	122			119
(2′-NAC)						
COOH			174			174
α	4.37	dd, J = 4.2, 4.6	54.5	4.32	dd, J = 4.4, 4.4	54.0
β	3.20	dd, J = 8.4, 14.0	37.0	3.40	dd, J = 4.4, 13.6	38.3
β	3.02	dd, J = 8.4, 13.6	37.0	3.01	dd, J = 8.6, 10.6	38.3
<u>C</u>OMe			172			173
CO<u>Me</u>	1.84[a]	s	22.4	1.79[b]	s	22.4
(5′-NAC)						
COOH			174			174
α	4.60	dd, J = 4.2, 4.2	53.9	4.53	dd, J = 4.6, 7.8	54.0
β	3.47	dd, J = 4.4, 14.4	35.8	3.45	dd, J = 4.4, 14.0	36.9
β	3.36	dd, J = 4.4, 14.0	35.8	3.30	m	36.9
<u>C</u>OMe			172			172
CO<u>Me</u>	1.95[a]	s	22.4	1.82[b]	s	22.4
(6′-NAC)						
COOH						174
α				4.28	dd, J = 4.0, 8.4	54.0
β				3.30	m	39.5
β				3.05	dd, J = 9.2, 10.0	39.5
<u>C</u>OMe						173
CO<u>Me</u>				1.97[b]	s	22.4
-O<u>Me</u>	3.82	s	55.8	3.82	s	55.8
-O<u>Me</u>	3.82	s	55.8	3.83	s	55.8

a, b: interchangeable within the same sign.

Figure S1. ^{1}H-NMR spectrum of TriNAC-PTS-catechol (**6**).

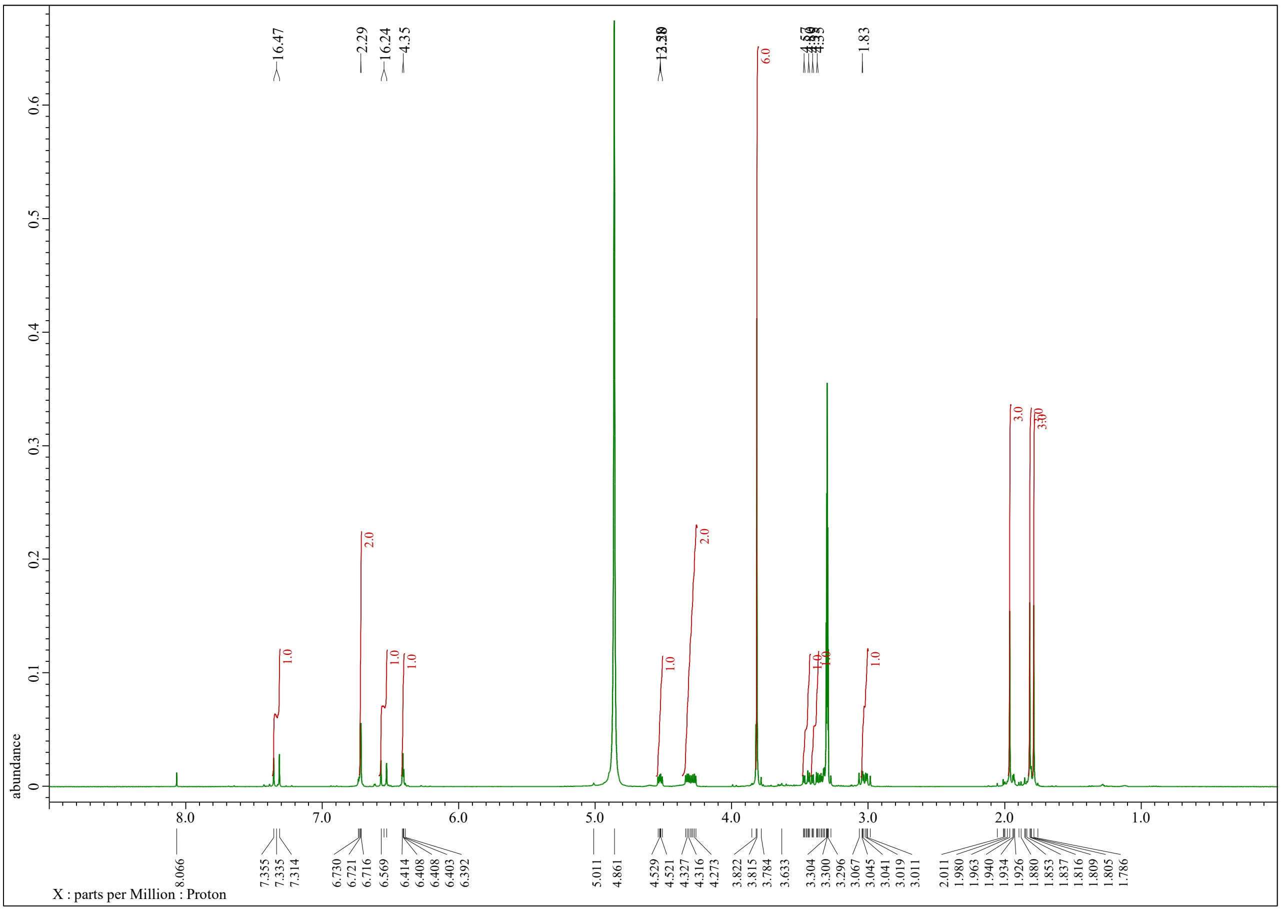

Figure S2. ^{1}H-NMR spectrum of DiNAC-PTS-catechol (**5**).

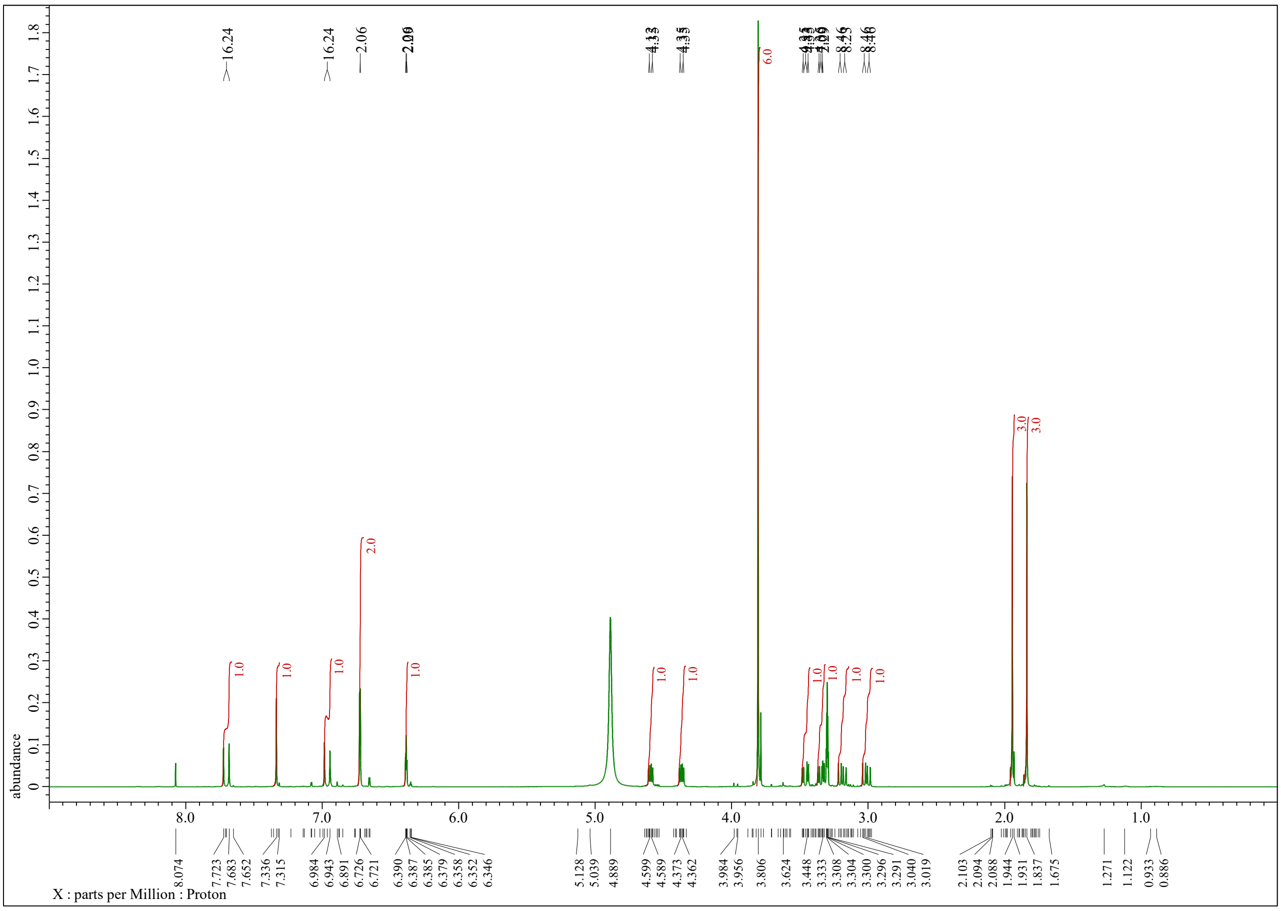

Figure S3. ^{1}H-NMR spectrum of PTS-catechol (**4**).

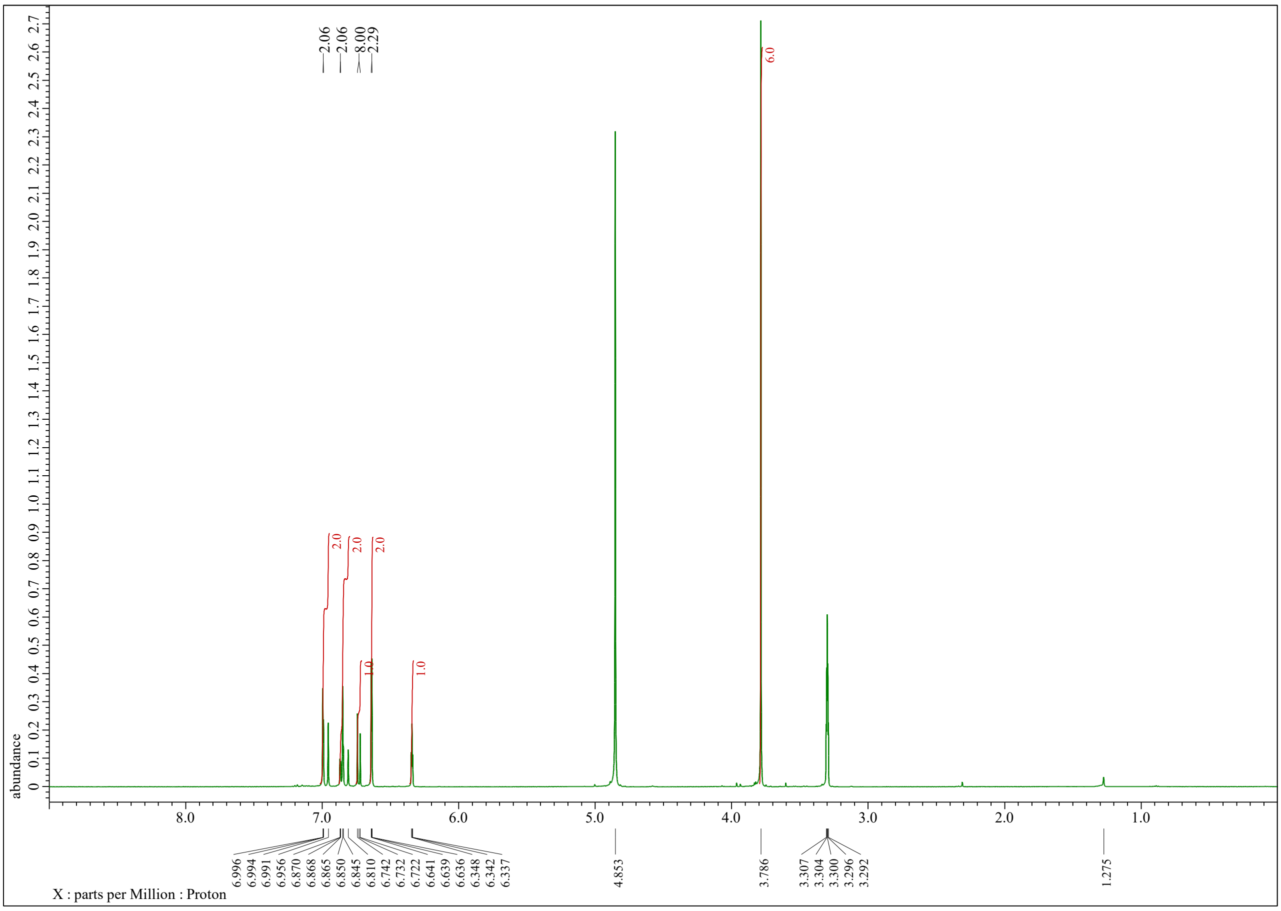

Figure S4. ^{13}C-NMR spectrum of DiNAC-PTS-catechol (**5**).

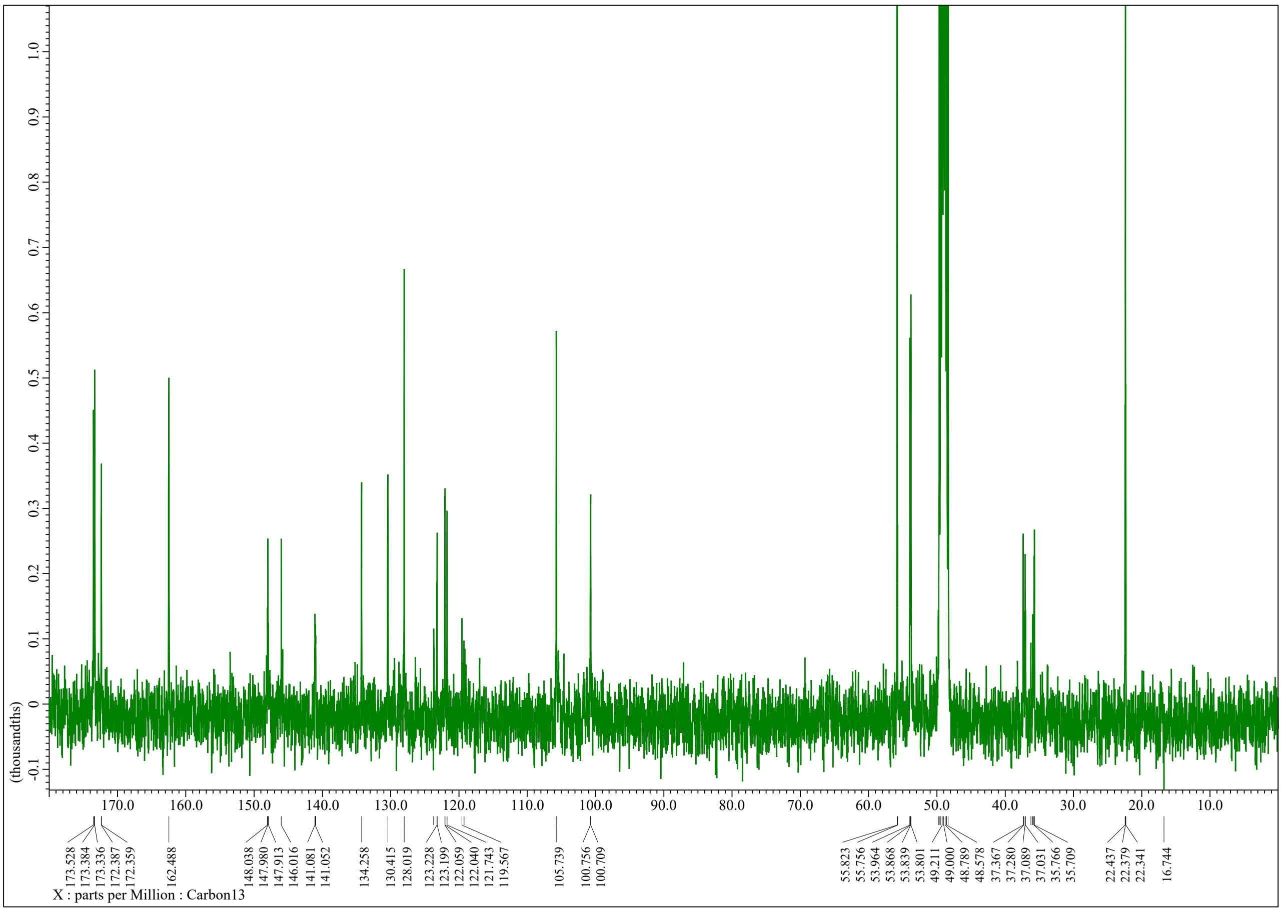

Figure S5. ^{13}C-NMR spectrum of TriNAC-PTS-catechol (**6**).

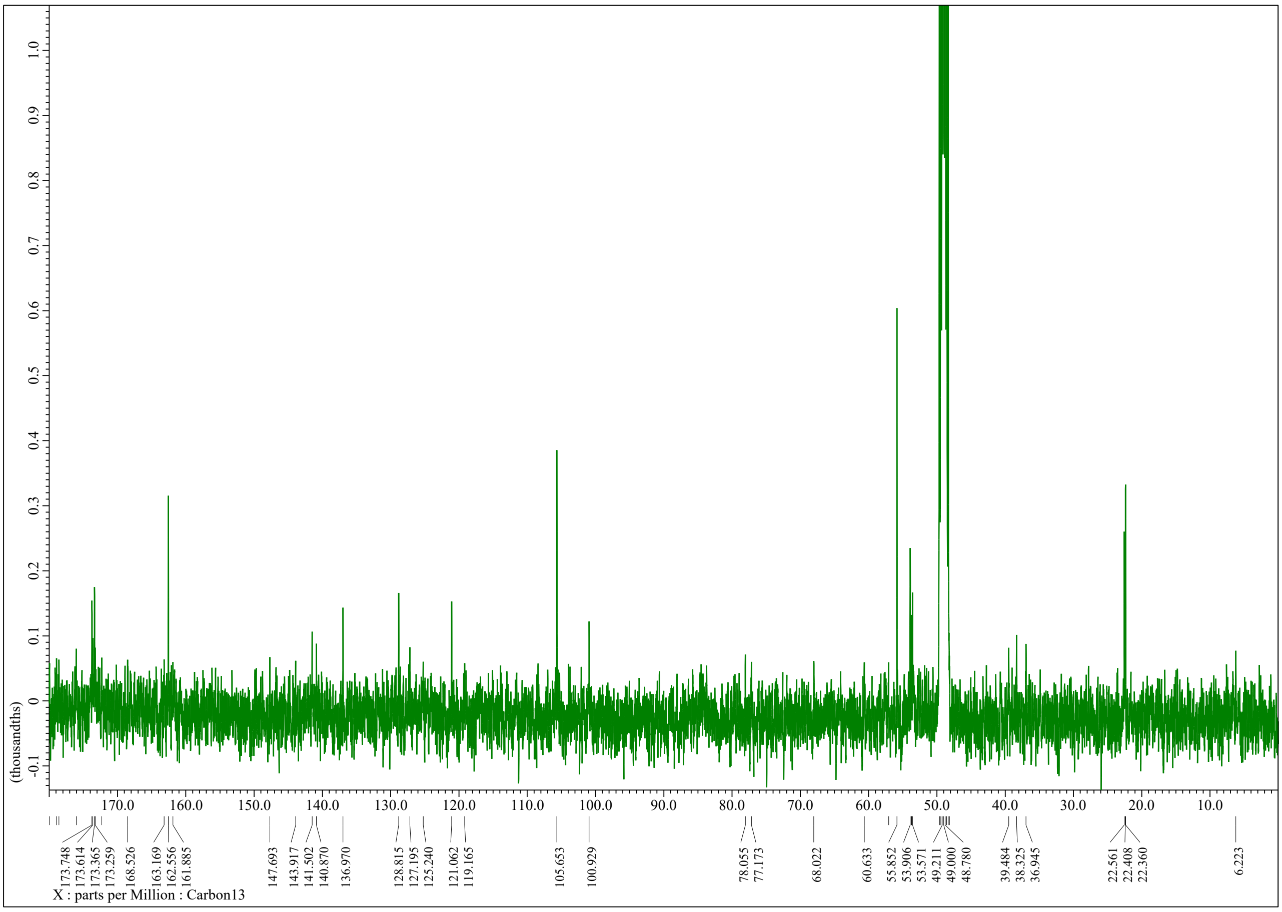

Figure S6. ^{13}C-NMR of PTS-catechol (**4**).

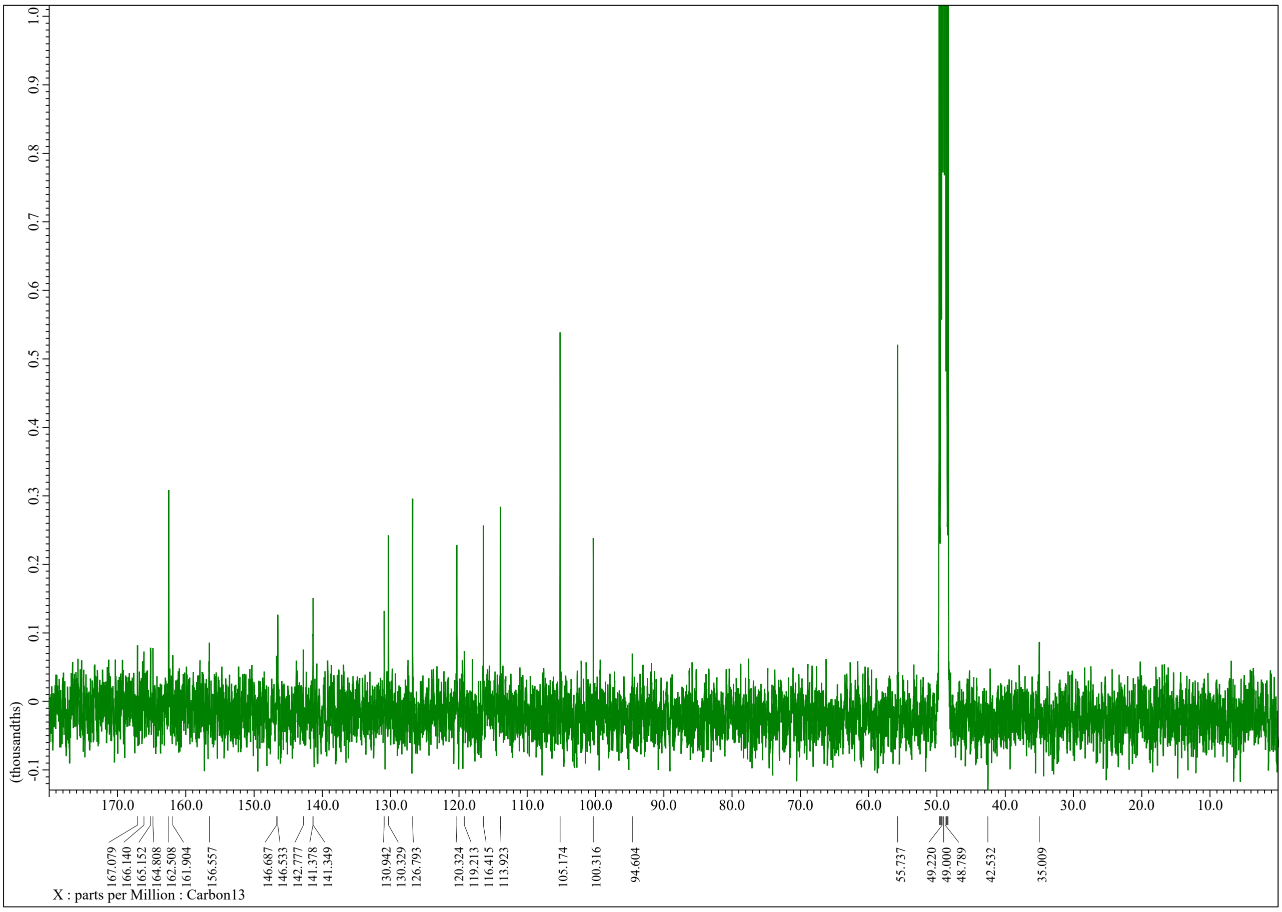

Figure S7. COSY of DiNAC-PTS-catechol (**5**).

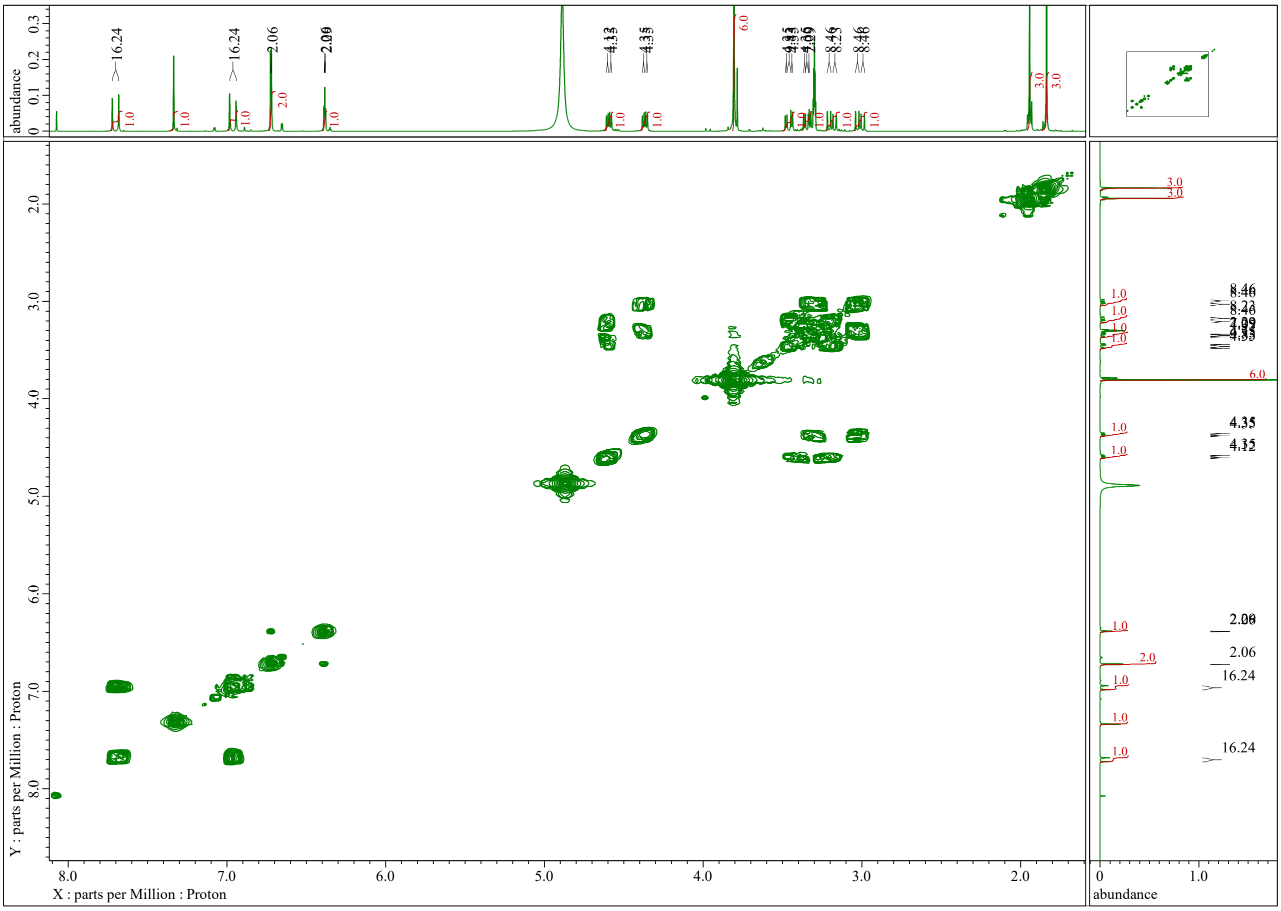

Figure S8. COSY of TriNAC-PTS-catechol (**6**).

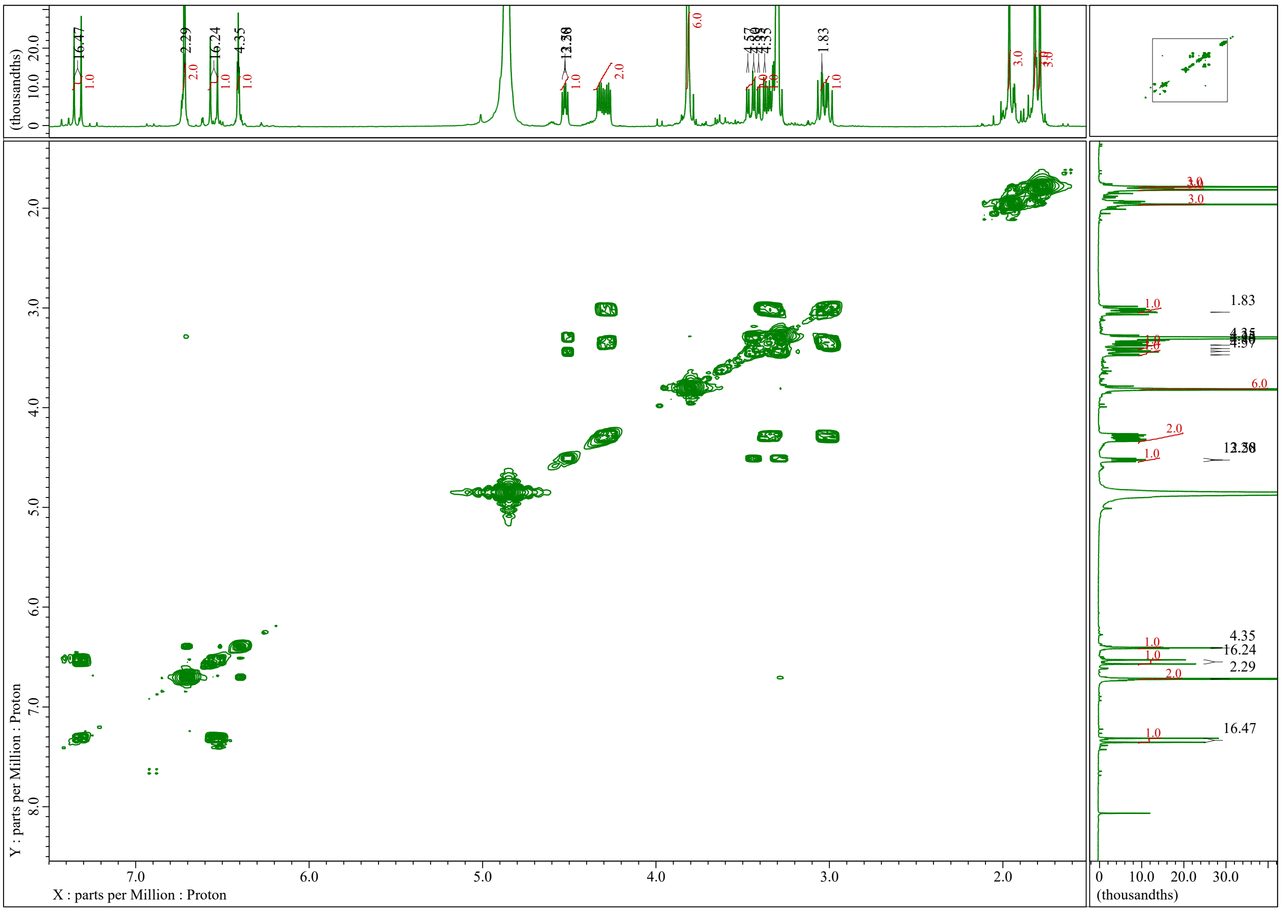

Figure S9. COSY of PTS-catechol (**4**).

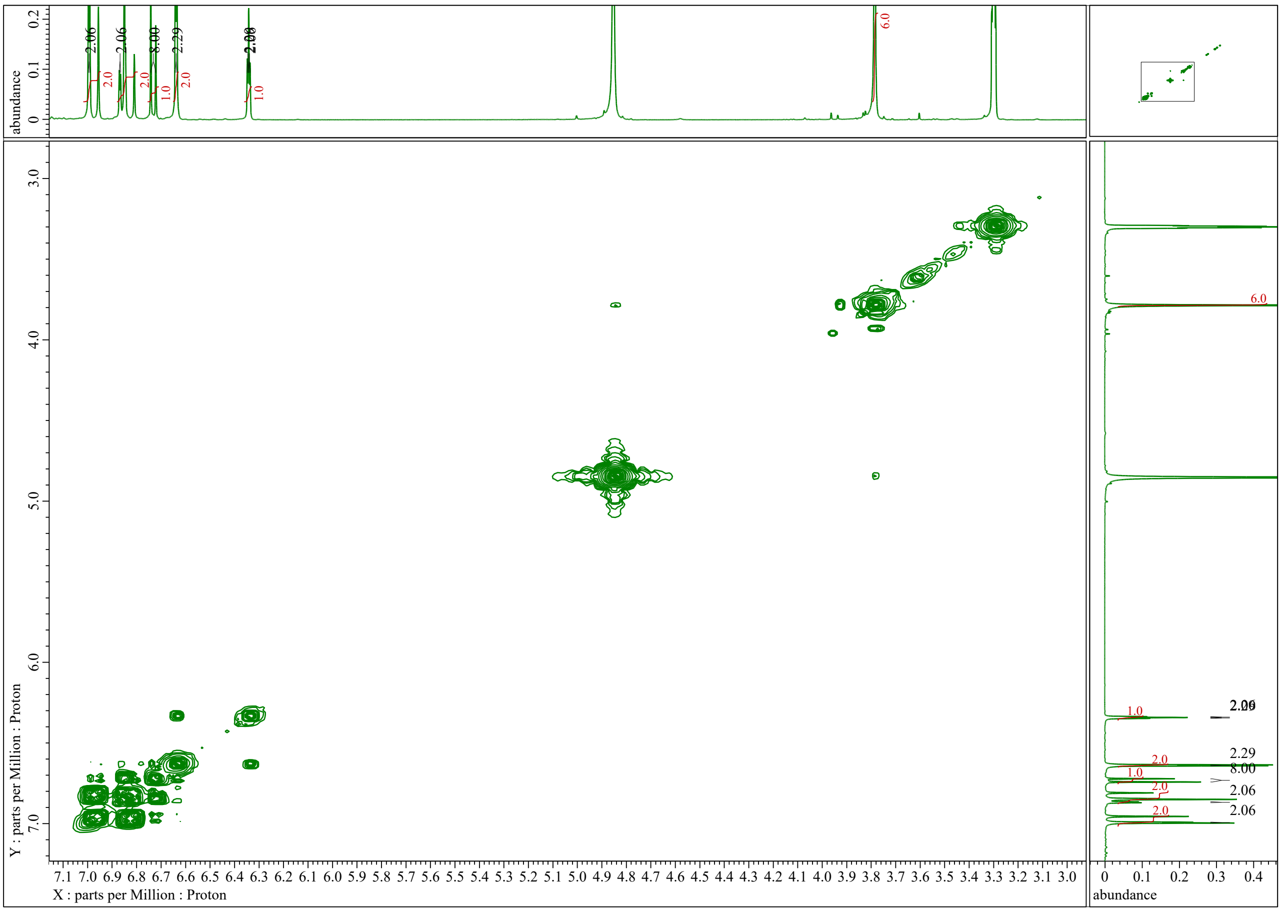

Figure S10. HMBC of DiNAC-PTS-catechol (**5**).

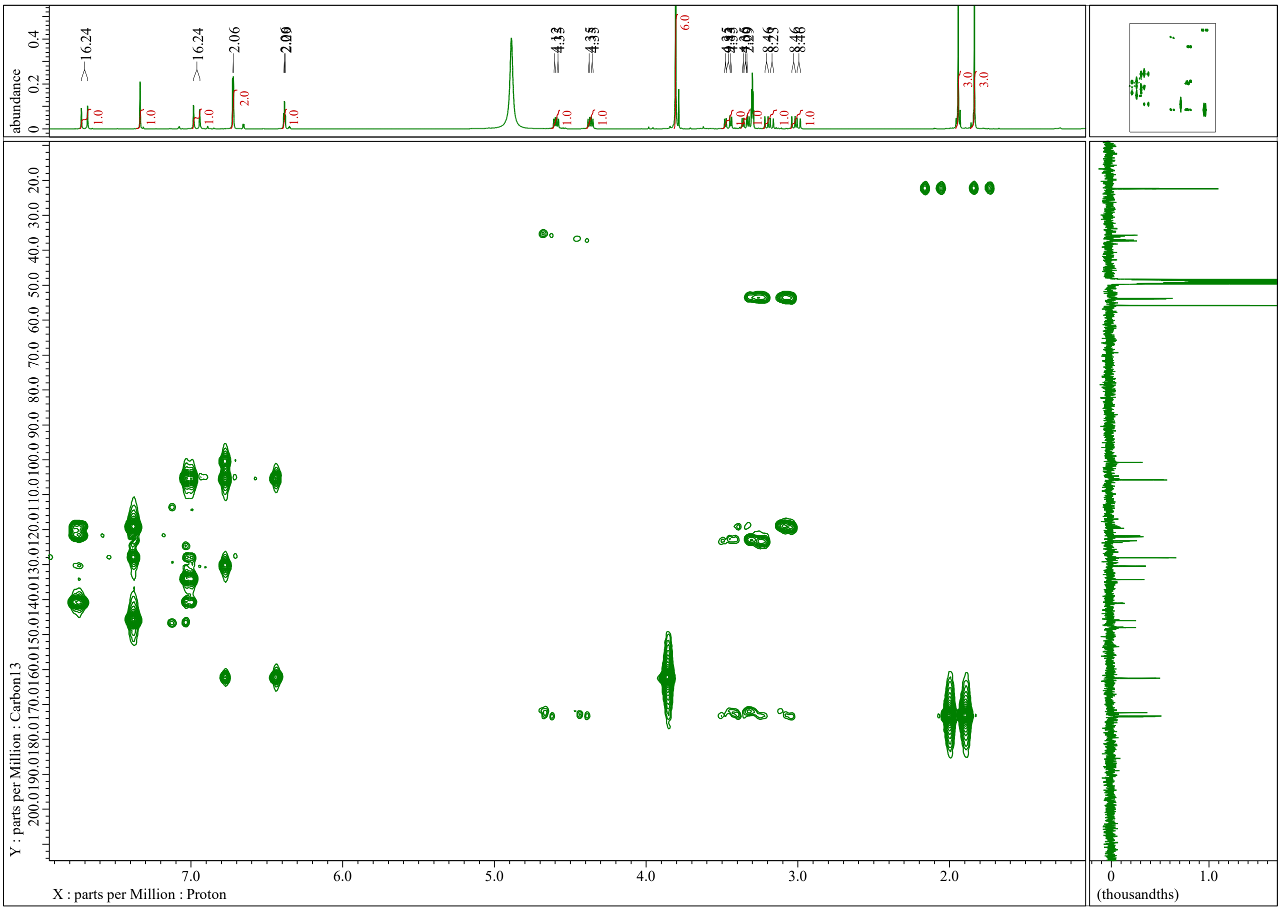

Figure S11. HMBC of TriNAC-PTS-catechol (**6**).

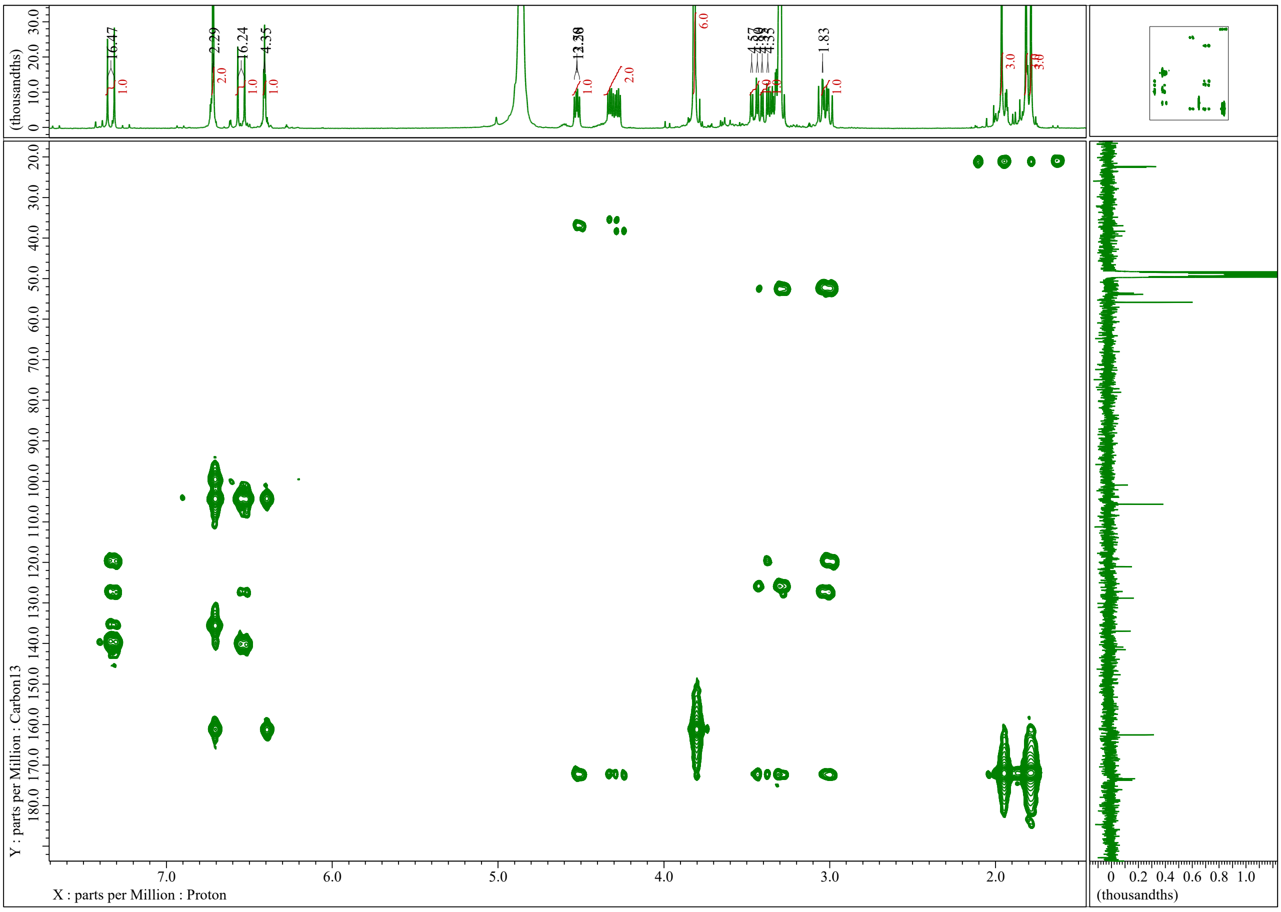

Figure S12. HMBC of PTS-catechol (**4**).

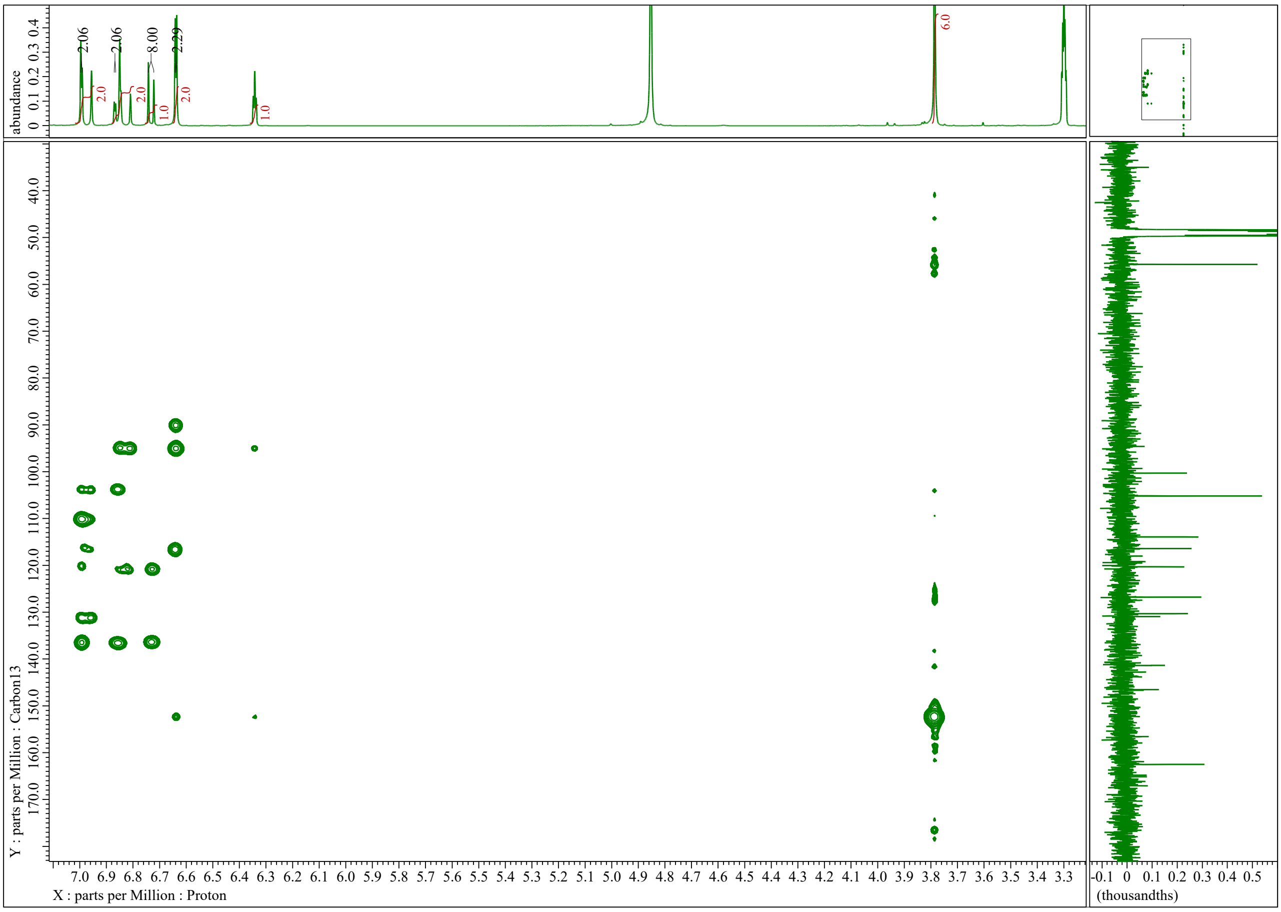

Figure S13. HMQC of DiNAC-PTS-catechol (**5**).

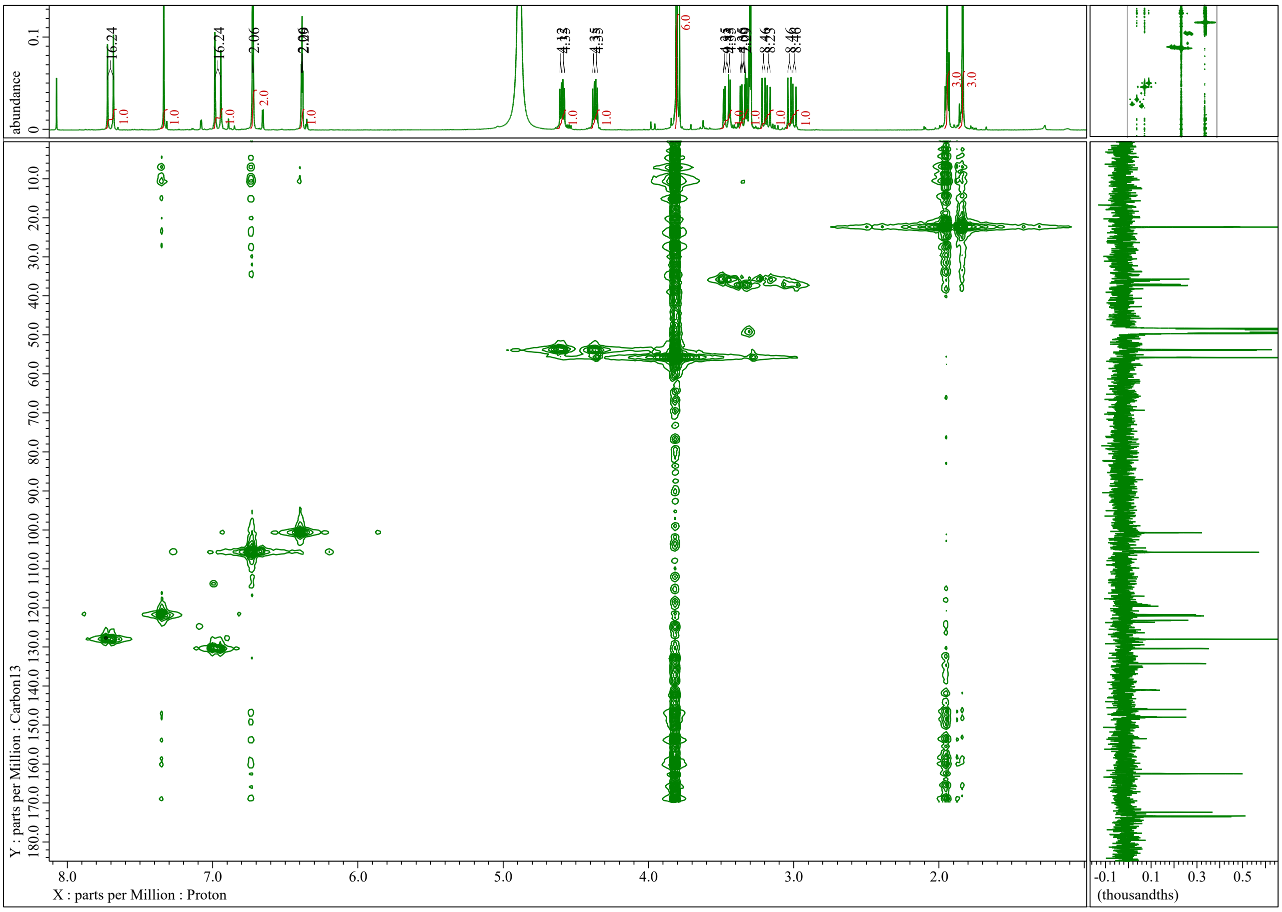

Figure S14. HMQC of TriNAC-PTS-catechol (**6**).

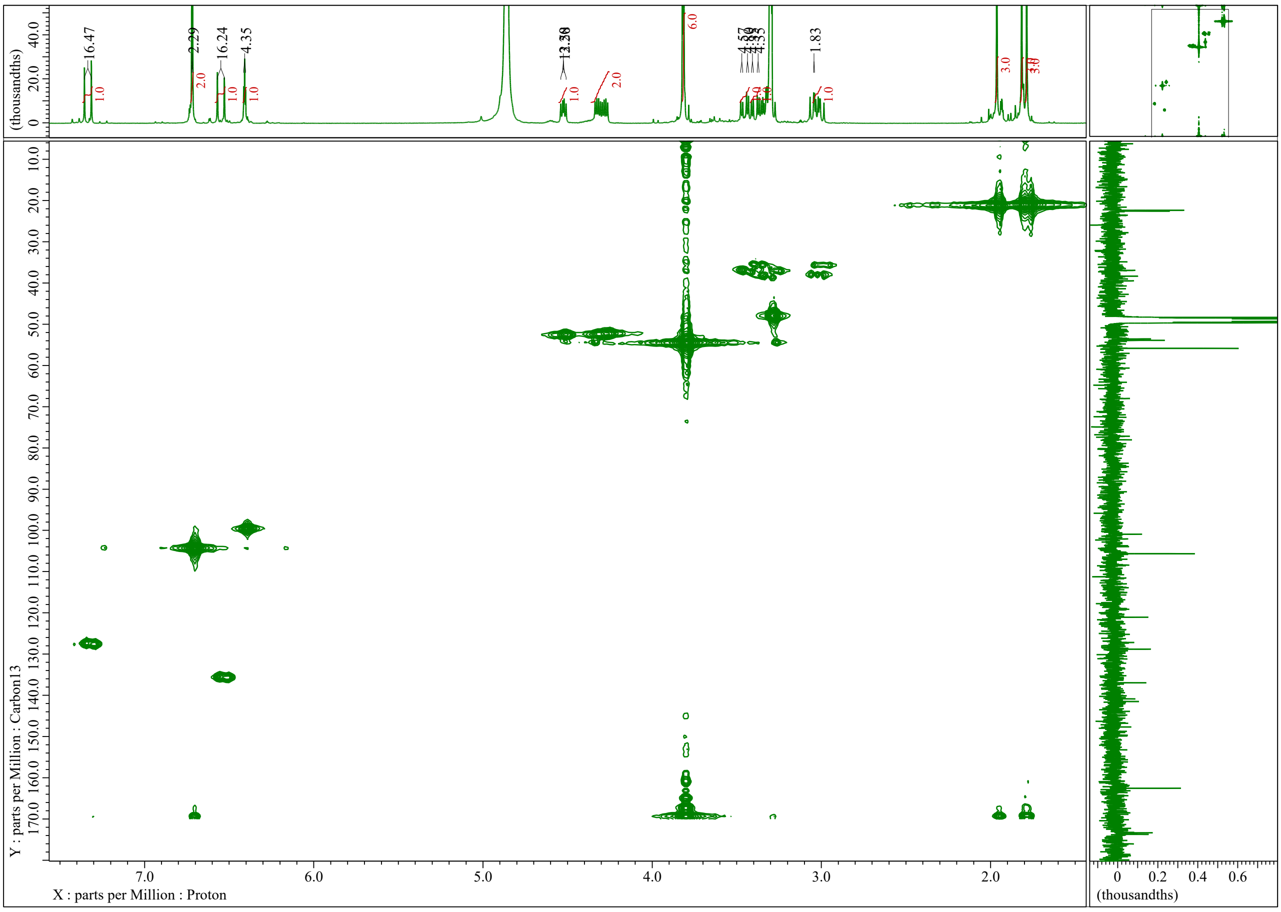

Figure S15. HMQC of PTS-catechol (**4**).

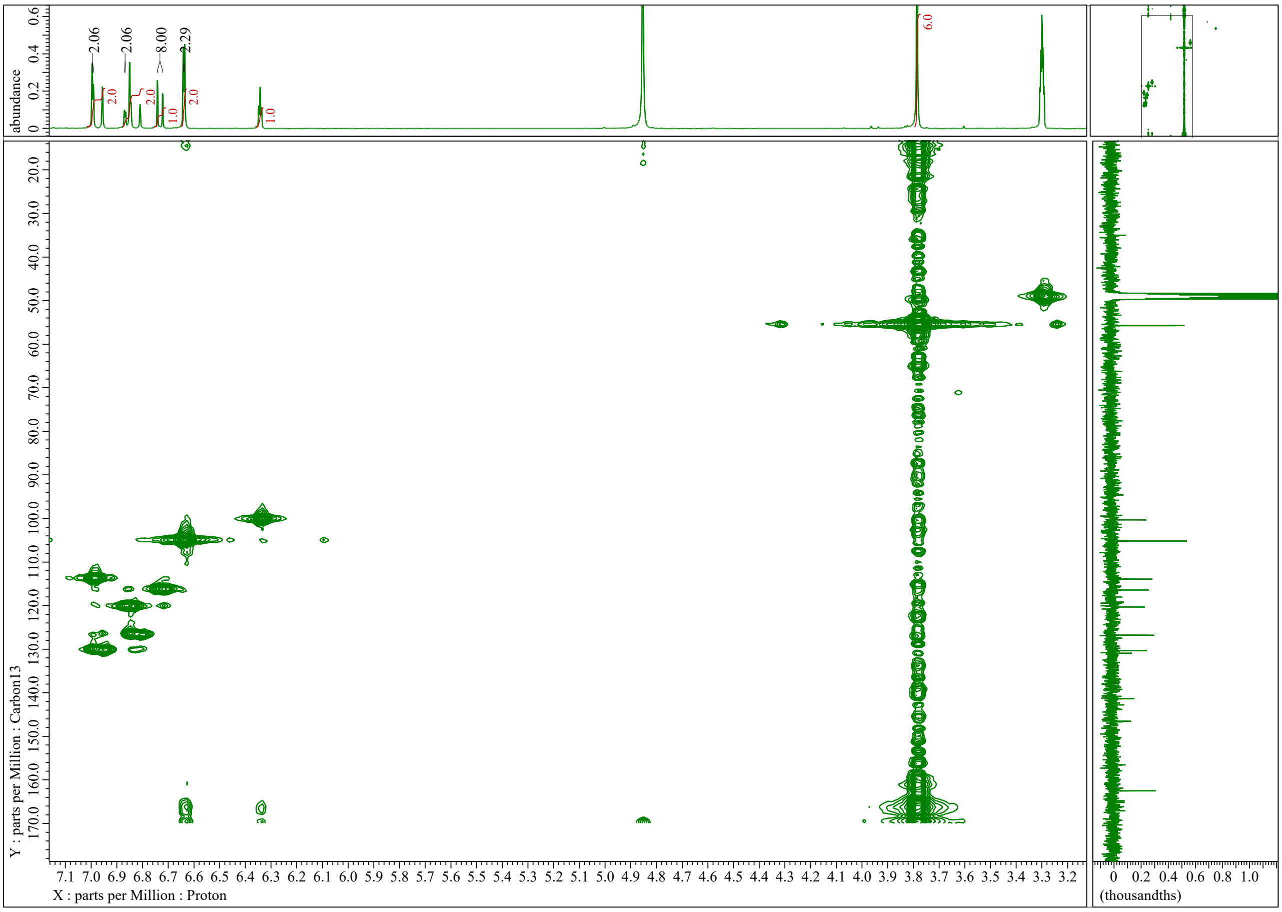

Figure S16. Tyrosinase-dependent cytotoxicity against **(a)** PTS (**2**), **(b)** RES (**1**), and **(c)** 4SCAP in human tyrosinase-expressing 293T cells (hTYR-293T cells). Viability of TYR- or mock-transfected cells treated with the indicated concentrations of compounds for 2 h were assessed. Data represent means ± SD (n = 3 wells). Results are representative of two or three independent experiments.

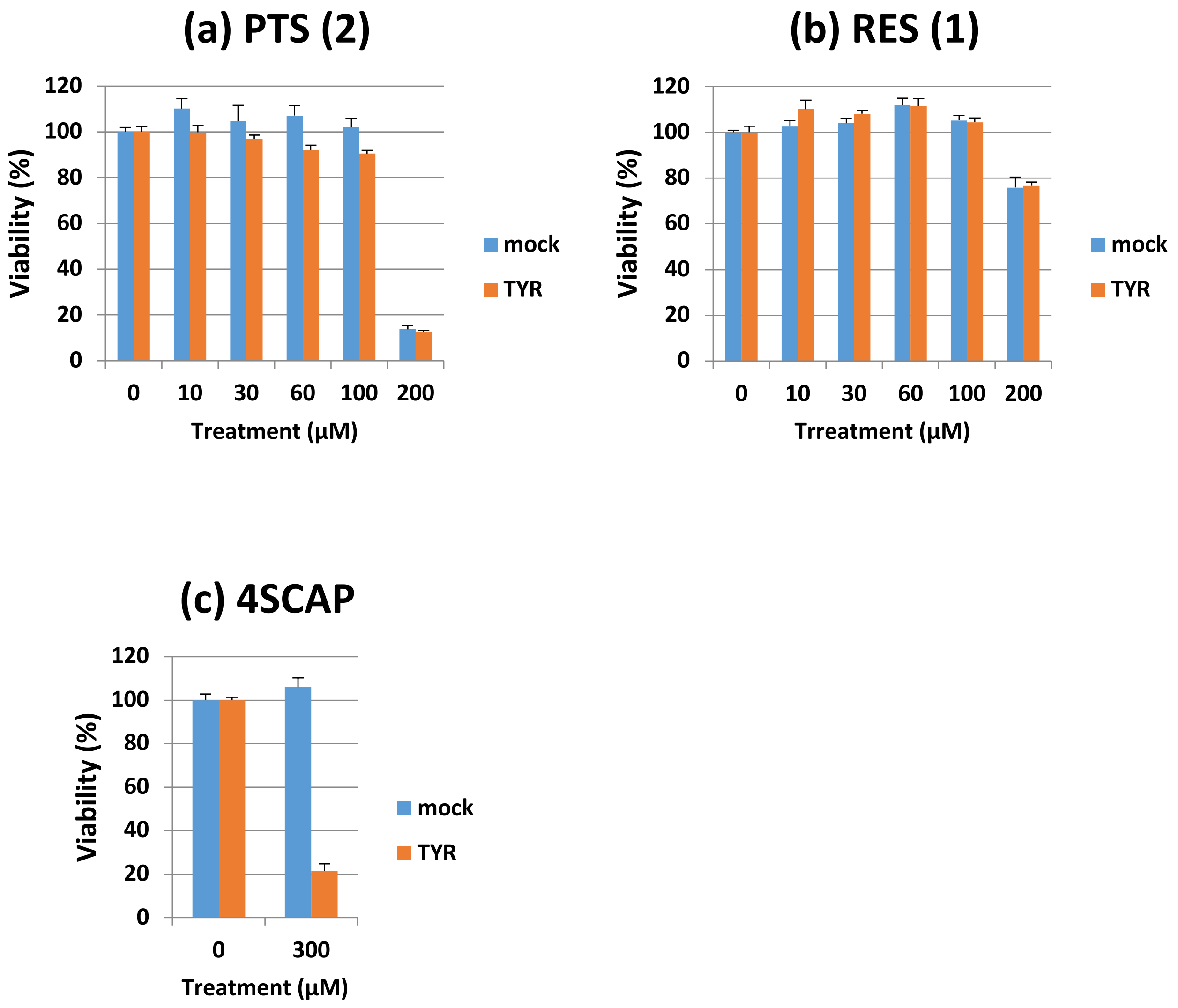

Supplement: Supplementary file 1 [file ijms-25-09990-s001.zip › ijms-3195220-supplementary.pdf]
